# Supplementary material for: Structural insights into Arabidopsis ethylene response factor 96 with an extended N-terminal binding to GCC box
Source: Plant Mol Biol. 2020 Aug 19;104(4):483–98. doi: 10.1007/s11103-020-01052-5 (PMC7593309; doi:10.1007/s11103-020-01052-5)
Supplement: Supplementary file 1 — Supplementary file1 (DOCX 62599 kb) [file 11103_2020_1052_MOESM1_ESM.docx]

**Structural insights into *Arabidopsis* ETHYLENE RESPONSE FACTOR 96 with an extended N-terminal binding to GCC box**

**Chun-Yen Chen^1^, Pei-Hsuan Lin^1^, Kun-Hung Chen^1^ and Yi-Sheng Cheng^1,2,3,*^**

^1^ Institute of Plant Biology, ^2^ Department of Life Science, ^3^ Genome and Systems Biology Degree Program, National Taiwan University, Taipei, Taiwan

***Correspondence**:

Dr. Yi-Sheng Cheng

[chengys@ntu.edu.tw](mailto:chengys@ntu.edu.tw)

**Supplementary Data**

**SUPPLEMENTARY TABLES AND FIGURE LEGEND**

**Supplementary Table 1.** List of DNA probe sequences.

**Supplementary Table 2.** Statistics of the intermolecular binding between the AtERF96 protein and GCC11 DNA motif.

**Supplementary Table 3.** Parameters of fluorescence polarization assay between GCC12 probes and various AtERF96 proteins.

**Supplementary Table 4.** Parameters of fluorescence polarization assay between AtERF96 proteins and various DNA motifs.

**Supplementary Table 5.** List of DNA primers used in the experiments

**Supplementary Figure 1.** Purification and crystallization of AtERF96 recombinant protein. (**A**) Size-exclusion chromatography trace of AtERF96 protein. AtERF96 was eluted as double peaks corresponding to aggregated proteins (41.5 mL) and following monomeric proteins (62.7 mL). (**B**) Purification and characterization of AtERF96 protein. M, protein marker. S, the supernatant of protein crude extract. P, the insoluble pellet. FT, the flow-through proteins of FPLC purification. E, the eluate of Ni-Sepharose column. H, the eluate of Heparin-Sepharose column. FP, the AtERF96 protein after buffer exchange by desalting column. (**C**) The hexagonal prism crystals of the AtERF96 protein were obtained after six-month growth. (**D and E**) The molecular weight of aggregated and monomeric AtERF96 proteins obtained from Size-exclusion chromatography was determined as 248 kDa (**D**) and 17.8 kDa (**E**) by dynamic light scattering analysis.

**Supplementary Figure 2.** The structural comparison of the AtERF96 and the AP2/ERF domain of AtERF100. The crystal structure of AtERF96 could be superimposed on the NMR structure of AtERF100 with a backbone root-mean-square deviation of 1.31 Å across 55 Cα atoms in the AP2/ERF domains.

**Supplementary Figure 3.** The alignment of the group IX of AP2/ERF family. Aligned identical residues shared across sequence are highlighted in red vertical stripe, and the blue empty squares indicate high similarity region. The protein entry of UniProt used are listed as follows: O49515 (AtERF91), Q8LDC8 (AtERF92), Q8VYM0 (AtERF93), Q9LND1 (AtERF94), Q9LTC6 (AtERF95), Q9LSX0 (AtERF96), P93822 (AtERF97), Q9LTC5 (AtERF98), Q8L9K1 (AtERF99), O80337 (AtERF100), O80338 (AtERF101), O80341 (AtERF102), Q8VZ91 (AtERF103), Q9FKG1 (AtERF104), Q8VY90 (AtERF105), Q9LY05 (AtERF106), Q9FKG2 (AtERF107).

**Supplementary Figure 4.** Characterization of the interaction between AtERF96 proteins and GCC box motifs. (**A-C**) EMSA binding assay of various AtERF96 proteins with GCC8 (**A**), GCC10 (**B)**, and GCC11 (**C**) probes. (**D-F**) quantification of the relative binding level of various AtERF96 proteins with GCC8 (**D**), GCC10 (**E**), and GCC11 (**F**) probes determined by EMSA assay. All data are representative of three independent experiments and the error is calculated as standard deviation.

**Supplementary Figure 5.** Characterization of the interaction between AtERF96 proteins and GCC box motifs. (**A and B**) EMSA binding assay of various AtERF96 proteins with GCC12 (**A**) and GCC15 (**B**) probes. (**C and D**) quantification of the relative binding level of various AtERF96 proteins with GCC12 (**C**) and GCC15 (**D**) probes determined by EMSA assay. All data are representative of three independent experiments and the error is calculated as standard deviation.

**Supplementary Figure 6.** Electron density map of GCC box motif. (**A and B**) Minor groove (**A**) and major groove (**B**) views of the GCC11 DNA motif with nucleotides C7 to G16 from the structure of AtERF96–GCC11 complex are contoured at the 1.5 *σ* of 2 *F*_o_–*F*_c_ map.

**Supplementary Figure 7.** Characterization of binding ability between AtERF96 proteins and various DNA motifs. (**A**) EMSA binding assay of AtERF96 wild type, R19A/R21A, and R31A/R39A proteins with the GCC12 probe. (**B**) Comparison of the AtERF96 binding ability between the GCC12 probe and W-box motif. (**C**) Competition assay by AtERF96 with fGCC12 labelled probe and various GCC12 non-labelled probe. Lane F: fGCC12 free probe. Lane 1: AtERF96 binding with fGCC12 labelled probe. Lane 2-5: the molar ratio of fGCC12 vs GCC12 is from 1:1 in Lane 2, 1:2 in Lane 3, 1:4 in Lane 4, and 1:8 in Lane 5.

**Supplementary Table 1.** List of DNA probe sequences.

| Probe name | Sequence | Modification |
| --- | --- | --- |
| GCC8 | AGCCGCCC | 5’-Fluorescein |
| GCC10 | AGAGCCGCCA | 5’-Fluorescein |
| GCC11 | TAGCCGCCAGC | 5’-Fluorescein |
| GCC12 | CCAGCCGCCCAT | 5’-Fluorescein |
| GCC15 | ATCGAGCCGCCATCG | 5’-Fluorescein |
| P box | CCGCCCTCCA | 5’-Fluorescein |
| CS1 box | TAGACCGCCT | 5’-Fluorescein |
| DRE box | AAAATTTCATGGCCGACCTGCTTTTAAGCT | 3’-Fluorescein |
| W box | TTAACTTGACCCAA | 5’-Fluorescein |

^*^ The underlined word refers to the core sequence in the DNA motifs.

**Supplementary Table 2.** Statistics of the intermolecular binding between the AtERF96 protein and GCC11 DNA motif.

| **No.** | **AtERF96** | **Dist. [Å]** | **GCC11** |
| --- | --- | --- | --- |
| 1 | A:GLN   3[ OE1] | 3.82 | B:DG  10[ N2 ] |
| 2 | A:ARG   6[ NH1] | 2.09 | B:DG  10[ OP1] |
| 3 | A:ARG  19[ NH2] | 2.46 | B:DG   3[ O6 ] |
| 4 | A:ARG  21[ NH1] | 3.82 | B:DT   1[ O4 ] |
| 5 | A:ARG  21[ NH2] | 2.72 | B:DG   3[ O6 ] |
| 6 | A:GLU  29[ OE1] | 2.27 | B:DC   5[ N4 ] |
| 7 | A:ARG  39[ NH1] | 2.85 | B:DG   6[ O6 ] |
| 8 | A:ASP   2[ N  ] | 3.88 | C:DG  16[ OP1] |
| 9 | A:GLN   3[ NE2] | 2.53 | C:DT  14[ O2 ] |
| 10 | A:LYS  14[ NZ ] | 2.44 | C:DC  17[ OP1] |
| 11 | A:ARG  16[ NH1] | 2.00 | C:DG  15[ OP1] |
| 12 | A:VAL  18[ N  ] | 3.49 | C:DG  16[ OP2] |
| 13 | A:ARG  19[ NH1] | 2.08 | C:DG  18[ O6 ] |
| 14 | A:ARG  19[ NH2] | 2.14 | C:DG  19[ O6 ] |
| 15 | A:ARG  20[ N  ] | 3.75 | C:DC  17[ OP2] |
| 16 | A:ARG  21[ NH2] | 3.75 | C:DG  19[ O6 ] |
| 17 | A:ARG  31[ N  ] | 3.74 | C:DG  15[ OP2] |
| 18 | A:ARG  31[ NH1] | 2.30 | C:DG  15[ O5'] |
| 19 | A:ARG  39[ NH1] | 2.30 | C:DG  16[ O6 ] |
| 20 | A:ARG  39[ NH2] | 2.15 | C:DG  15[ O6 ] |
| 21 | A:LYS 131[ NZ ] | 3.14 | C:DC  13[ OP1] |

^*^ The bound residues and distances are determined by PISA server.

**Supplementary Table 3.** Parameters of fluorescence polarization assay between GCC12 probes and various AtERF96 proteins.

|  | Protein conc. [μM] | B_max1_ | K_d1_[μM] | K_d1_ Relative level | B_max2_ | K_d2_[μM] | K_d2_ Relative level | R^2^ |
| --- | --- | --- | --- | --- | --- | --- | --- | --- |
| ERF96 | 112.35 | 0.08±0.003 | 0.06±0.01 | 1.00 | 0.16±0.005 | 10.73±1.36 | 1.00 | 0.99 |
| R16A | 112.35 | 0.09±0.003 | 0.08±0.01 | 1.33 | 0.17±0.010 | 37.82±7.01 | 3.53 | 0.97 |
| R19A | 112.35 | 0.21±0.003 | 19.94±1.21 | 332.33 | 0.06±0.002 | 0.10±0.01 | 0.01 | 0.99 |
| R21A | 112.35 | 0.12±0.010 | 20.66±6.81 | 344.33 | 0.11±0.012 | 0.64±0.14 | 0.06 | 0.98 |
| W23A | 112.35 | 0.08±0.003 | 0.001±0.0001 | 0.02 | 0.15±0.003 | 1.37±0.14 | 0.13 | 0.98 |
| R31A | 112.35 | 0.11±0.004 | 0.11±0.01 | 1.83 | 0.09±0.004 | 11.95±2.60 | 1.11 | 0.99 |
| R39A | 112.35 | 0.23±0.005 | 13.42±1.34 | 223.67 | 0.04±0.004 | 0.05±0.02 | 0.01 | 0.98 |
| W41A | 112.35 | 0.09±0.008 | 0.03±0.01 | 0.5 | 0.12±0.007 | 1.77±0.36 | 0.16 | 0.97 |
| R19A/R21A | 60.73 | 0.07±0.010 | 6.50±1.60 | 108.33 | 0.01±0.011 | 0.70 ±0.67 | 0.07 | 0.99 |
| R31A/R39A | 52.26 | 0.04±0.004 | 5.91±1.16 | 98.5 | ND | ND | - | 0.96 |

^*^ All data are representative of at least three independent experiments and the error is calculated as SD.

**Supplementary Table 4.** Parameters of fluorescence polarization assay between AtERF96 proteins and various DNA motifs.

| Probe [10 nM] | B_max1_ | K_d1_[μM] | Kd_1_ Relative level | B_max2_ | K_d2_[μM] | Kd_2_ Relative level | R^2^ |
| --- | --- | --- | --- | --- | --- | --- | --- |
| GCC12 | 0.08±0.004 | 0.06±0.01 | 1.00 | 0.16±0.005 | 10.73±1.36 | 1.00 | 0.99 |
| P box | 0.08±0.006 | 0.25±0.06 | 4.17 | 0.20±0.006 | 27.39±4.00 | 2.55 | 0.99 |
| CS1 box | 0.08±0.005 | 0.79±0.11 | 13.17 | 0.24±0.015 | 77.29±14.23 | 7.20 | 0.99 |
| DRE box | 0.03±0.009 | 0.16±0.13 | 2.67 | 0.11±0.008 | 6.93±1.65 | 0.65 | 0.98 |

^1^ All data are representative of at least three independent experiments and the error is calculated as SD.

^2^ The concentrations of all AtERF96 proteins are 112 μM.

**Supplementary Table 5.** List of DNA primers used in the experiments

| Primer name | Sequence |
| --- | --- |
| ERF96 F | 5’ ATGGATCAAGGAGGTCGAGG |
| ERF96 R | 5’ TCATTTCTTCTTGCCCTTGTTAGGTT |
| BamHI-ERF96 | 5' ATTGGATCCATGGATCAAGGAGGTCGAGGT |
| ERF96-EcoRI | \| 5’ CGCGAATTCTCATTTCTTCTTGCCCTTGTTAGG \| \| --- \| |
| R19A/R21A F | 5' CCGGGGAGTTGCTAGAGCCCCTTGGGGAAAATATGC 3' |
| R19A/R21A R | 5' GCATATTTTCCCCAAGGGGCTCTAGCAACTCCCCGG 3' |
| R31A/R39A F | 5' GAGGAAGCACGGTGAAGCGGTGTGGCTTGGAACG 3' |
| R31A/R39A R | 5' CGTTCCAAGCCACACCGCTTCACCGTGCTTCCTC 3' |


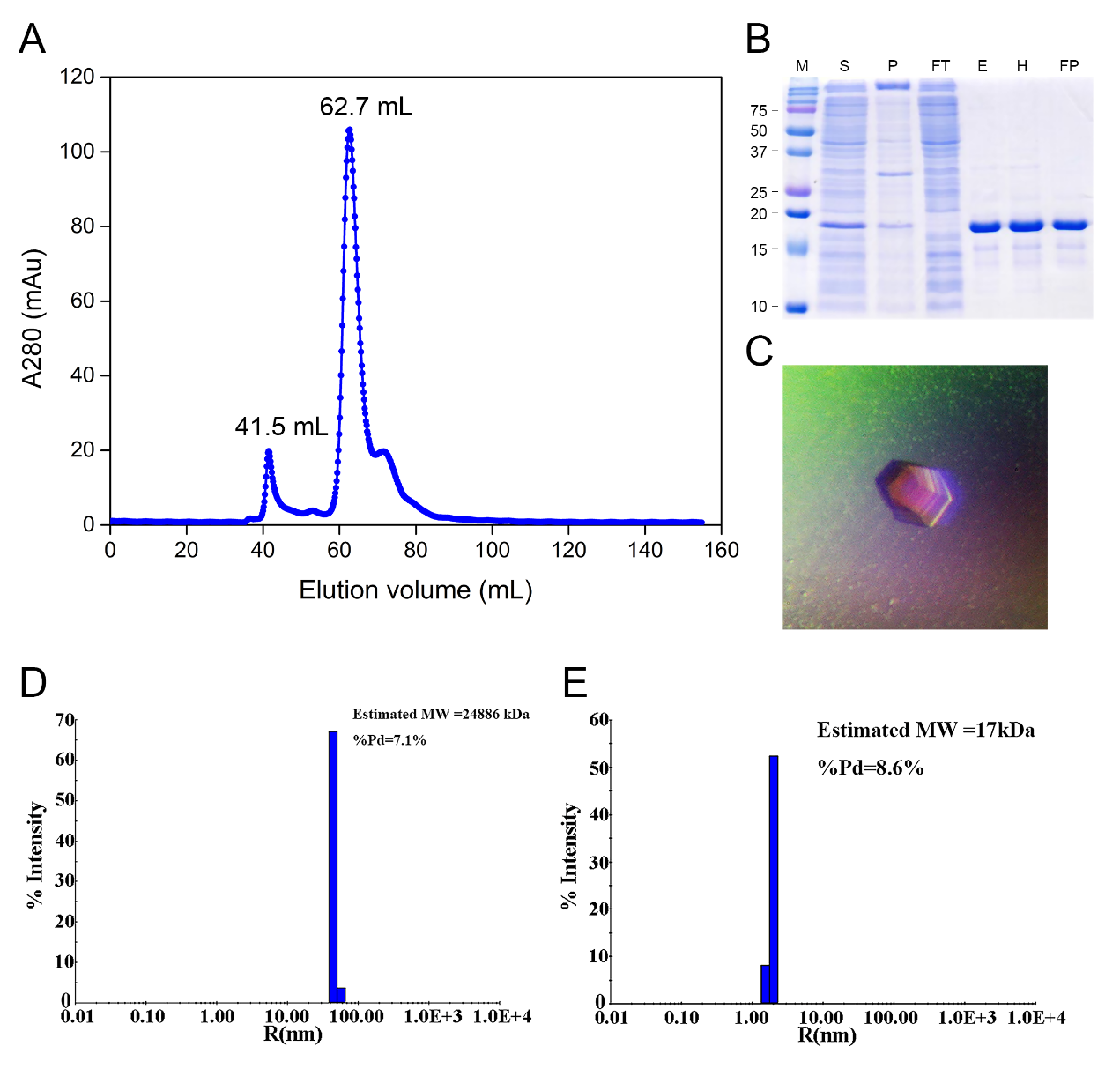


**Estimated MW=248.86 kDa**

**Supplementary Figure 1.** Purification and crystallization of AtERF96 recombinant protein. (**A**) Size-exclusion chromatography trace of AtERF96 protein. AtERF96 was eluted as double peaks corresponding to aggregated proteins (41.5 mL) and following monomeric proteins (62.7 mL). (**B**) Purification and characterization of AtERF96 protein. M, protein marker. S, the supernatant of protein crude extract. P, the insoluble pellet. FT, the flow-through proteins of FPLC purification. E, the eluate of Ni-Sepharose column. H, the eluate of Heparin-Sepharose column. FP, the AtERF96 protein after buffer exchange by desalting column. (**C**) The hexagonal prism crystals of the AtERF96 protein were obtained after six-month growth. (**D and E**) The molecular weight of aggregated and monomeric AtERF96 proteins obtained from Size-exclusion chromatography was determined as 248 kDa (**D**) and 17.8 kDa (**E**) by dynamic light scattering analysis.


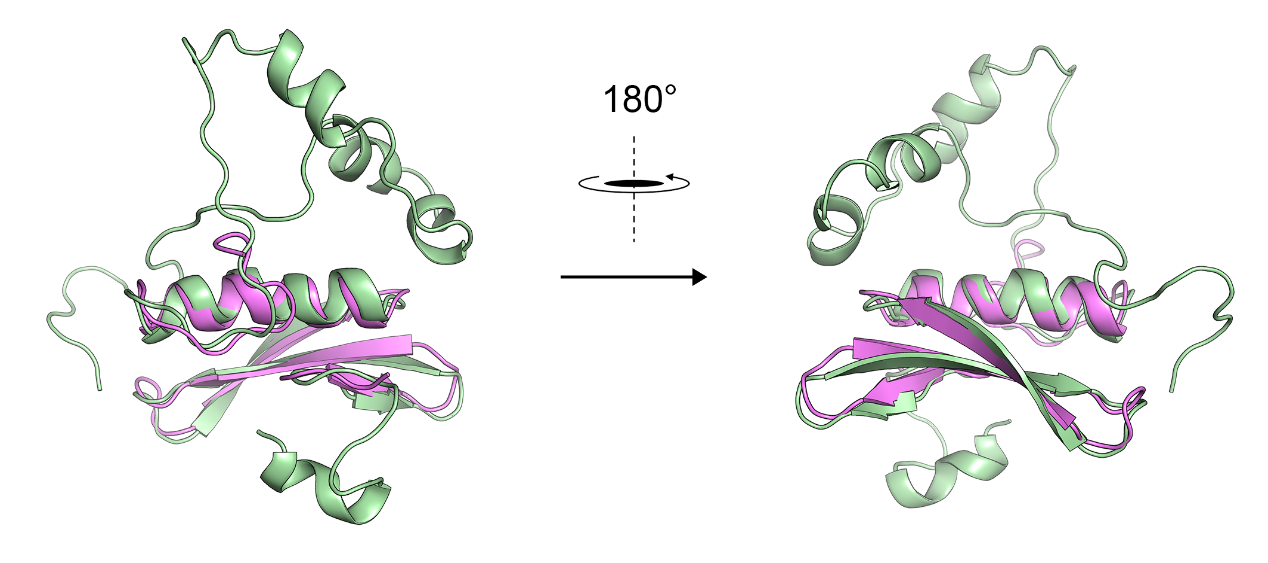


**Supplementary Figure 2.** The structural comparison of the AtERF96 and the AP2/ERF domain of AtERF100. The crystal structure of AtERF96 could be superimposed on the NMR structure of AtERF100 with a backbone root-mean-square deviation of 1.31 Å across 55 Cα atoms in the AP2/ERF domains.

**Supplementary Figure 3.** The alignment of the group IX of AP2/ERF family. Aligned identical residues shared across sequence are highlighted in red vertical stripe, and the blue empty squares indicate high similarity region. The protein entry of UniProt used are listed as follows: O49515 (AtERF91), Q8LDC8 (AtERF92), Q8VYM0 (AtERF93), Q9LND1 (AtERF94), Q9LTC6 (AtERF95), Q9LSX0 (AtERF96), P93822 (AtERF97), Q9LTC5 (AtERF98), Q8L9K1 (AtERF99), O80337 (AtERF100), O80338 (AtERF101), O80341 (AtERF102), Q8VZ91 (AtERF103), Q9FKG1 (AtERF104), Q8VY90 (AtERF105), Q9LY05 (AtERF106), Q9FKG2 (AtERF107).


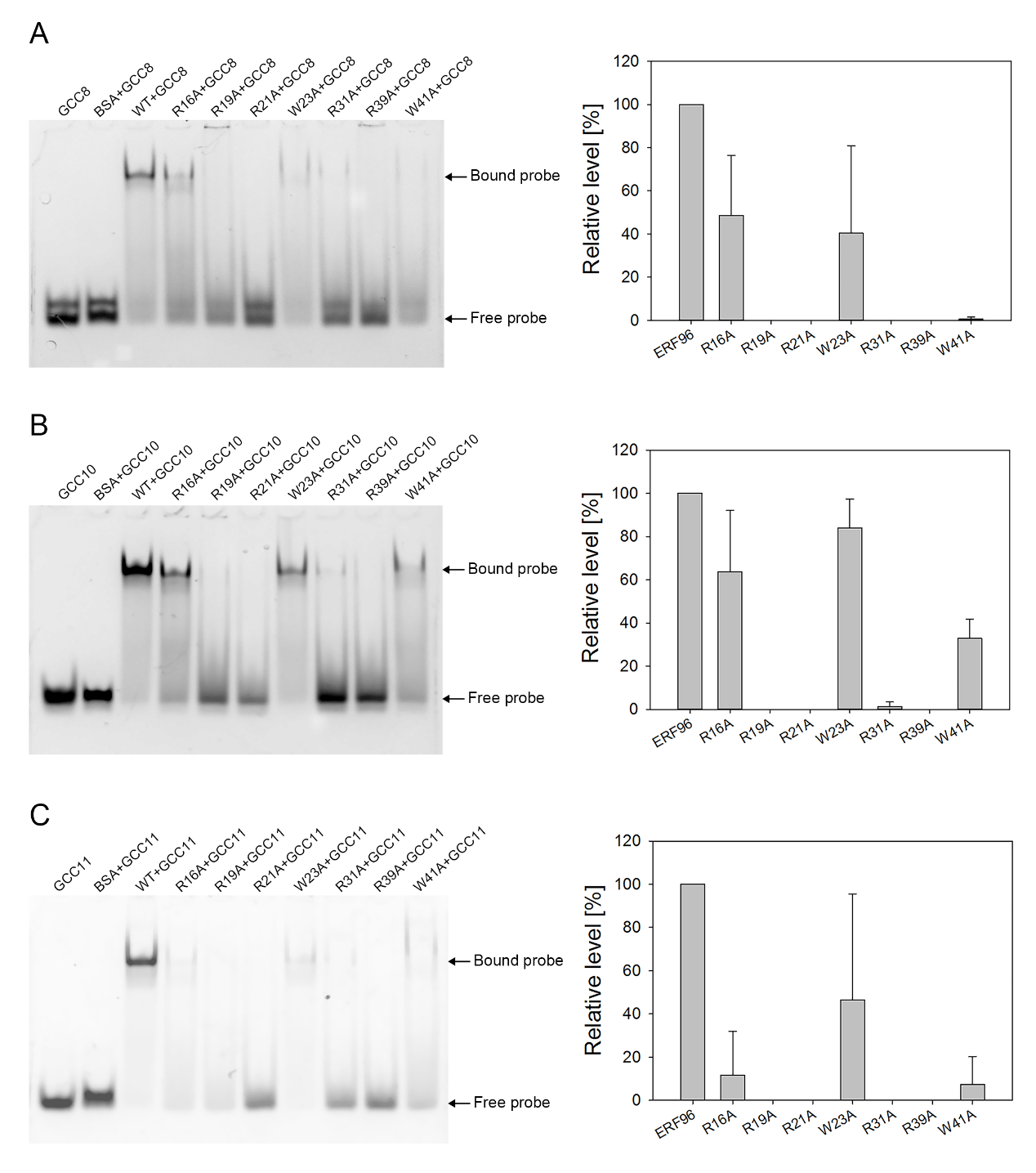


**Supplementary Figure 4.** Characterization of the interaction between AtERF96 proteins and GCC box motifs. (**A-C**) EMSA binding assay of various AtERF96 proteins with GCC8 (**A**), GCC10 (**B)**, and GCC11 (**C**) probes. (**D-F**) quantification of the relative binding level of various AtERF96 proteins with GCC8 (**D**), GCC10 (**E**), and GCC11 (**F**) probes determined by EMSA assay. All data are representative of three independent experiments and the error is calculated as standard deviation.


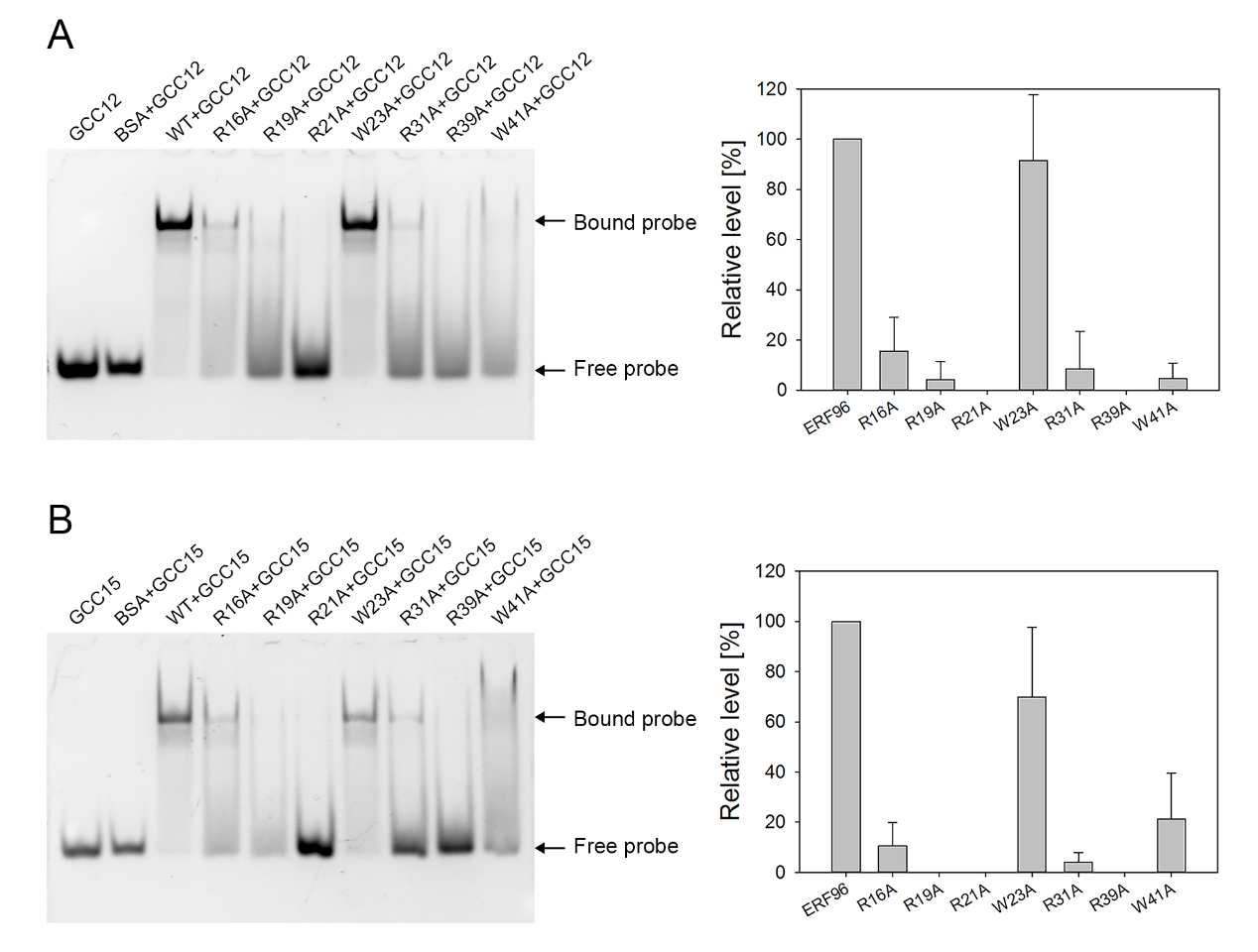


**Supplementary Figure 5.** Characterization of the interaction between AtERF96 proteins and GCC box motifs. (**A and B**) EMSA binding assay of various AtERF96 proteins with GCC12 (**A**) and GCC15 (**B**) probes. (**C and D**) quantification of the relative binding level of various AtERF96 proteins with GCC12 (**C**) and GCC15 (**D**) probes determined by EMSA assay. All data are representative of three independent experiments and the error is calculated as standard deviation.


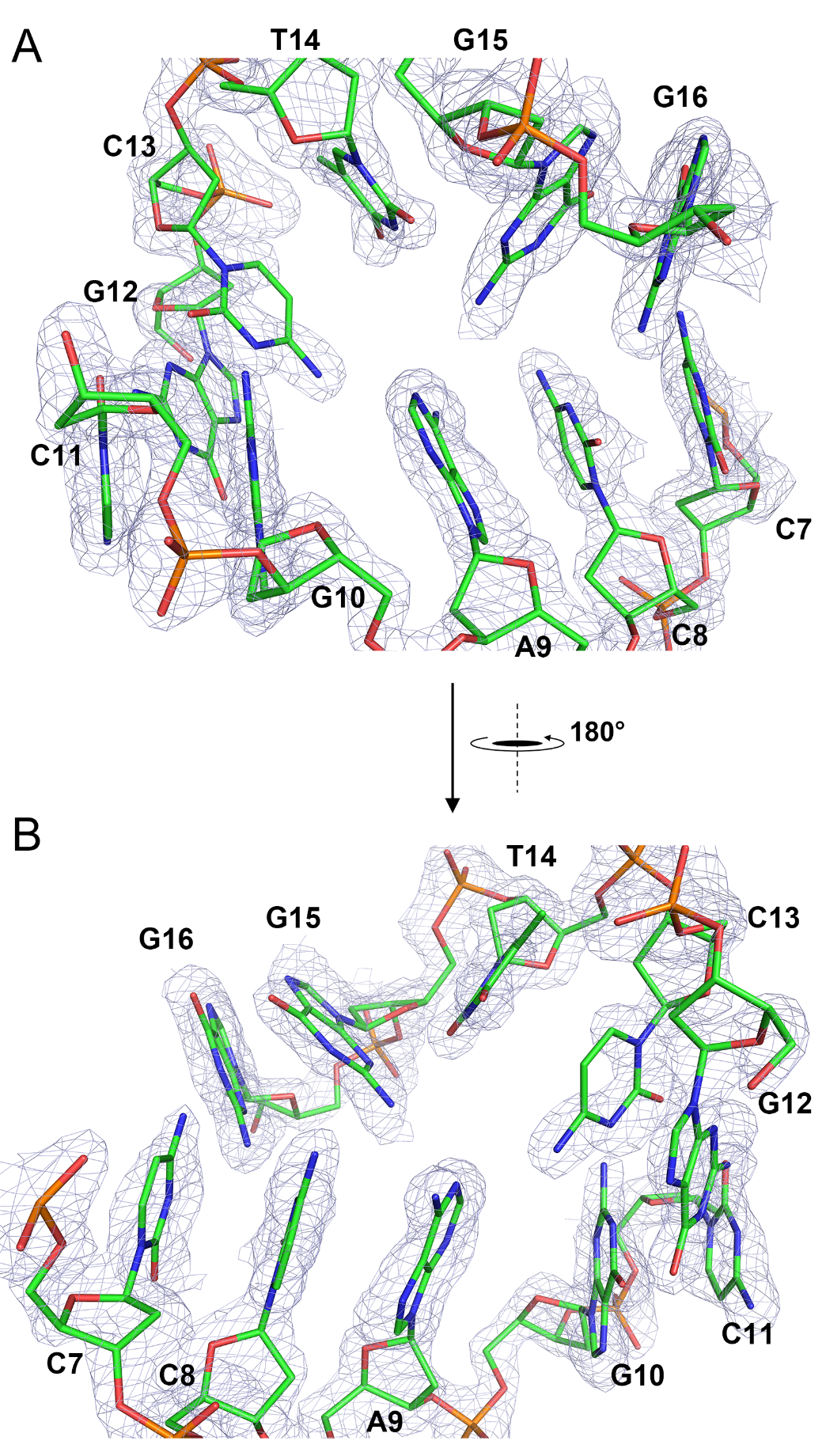


**Supplementary Figure 6.** Electron density map of GCC box motif. (**A and B**) Minor groove (**A**) and major groove (**B**) views of the GCC11 DNA motif with nucleotides C7 to G16 from the structure of AtERF96–GCC11 complex are contoured at the 1.5 *σ* of 2 *F*_o_–*F*_c_ map.


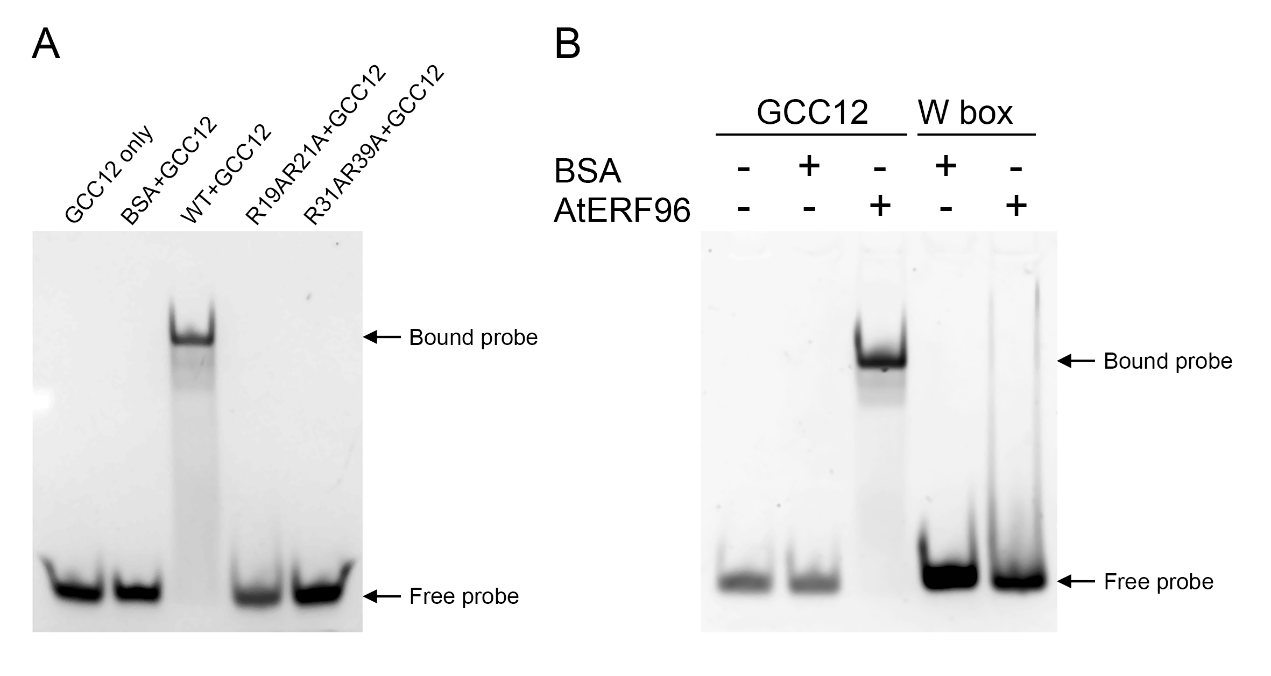


C


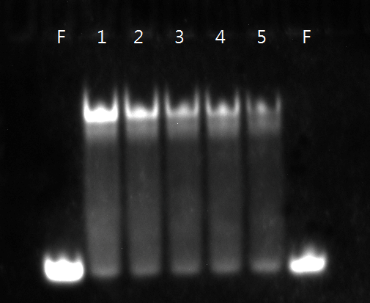


**Supplementary Figure 7.** Characterization of binding ability between AtERF96 proteins and various DNA motifs. (**A**) EMSA binding assay of AtERF96 wild type, R19A/R21A, and R31A/R39A proteins with the GCC12 probe. (**B**) Comparison of the AtERF96 binding ability between the GCC12 probe and W-box motif. (**C**) Competition assay by AtERF96 with fGCC12 labelled probe and various GCC12 non-labelled probe. Lane F: fGCC12 free probe. Lane 1: AtERF96 binding with fGCC12 labelled probe. Lane 2-5: AtERF96 with the molar ratio of fGCC12 vs GCC12 is from 1:1 in Lane 2, 1:2 in Lane 3, 1:4 in Lane 4, and 1:8 in Lane 5.
